# An Artificial Intelligence Model for Predicting 1-Year Survival of Bone Metastases in Non-Small-Cell Lung Cancer Patients Based on XGBoost Algorithm

**DOI:** 10.1155/2020/3462363

**Published:** 2020-06-27

**Authors:** Zhangheng Huang, Chuan Hu, Changxing Chi, Zhe Jiang, Yuexin Tong, Chengliang Zhao

**Affiliations:** ^1^Department of Spine Surgery, Affiliated Hospital of Chengde Medical University, Shuangqiao District, Chengde, Hebei Province, China; ^2^Department of Orthopedic, The Affiliated Hospital of Qingdao University, Shinan District, Qingdao, Shandong Province, China; ^3^Department of Radiotherapy, The Third Affiliated Hospital of Kunming Medical University, Chenggong District, Kunming, Yunnan Province, China; ^4^School of Public Health, Jilin University, Chaoyang District, Changchun, Jilin Province, China

## Abstract

Non-small-cell lung cancer (NSCLC) patients often develop bone metastases (BM), and the overall survival for these patients is usually perishing. However, a model with high accuracy for predicting the survival of NSCLC with BM is still lacking. Here, we aimed to establish a model based on artificial intelligence for predicting the 1-year survival rate of NSCLC with BM by using extreme gradient boosting (XGBoost), a large-scale machine learning algorithm. We selected NSCLC patients with BM between 2010 and 2015 from the Surveillance, Epidemiology, and End Results database. In total, 5973 cases were enrolled and divided into the training (*n* = 4183) and validation (*n* = 1790) sets. XGBoost, random forest, support vector machine, and logistic algorithms were used to generate predictive models. Receiver operating characteristic curves were used to evaluate and compare the predictive performance of each model. The parameters including tumor size, age, race, sex, primary site, histological subtype, grade, laterality, T stage, N stage, surgery, radiotherapy, chemotherapy, distant metastases to other sites (lung, brain, and liver), and marital status were selected to construct all predictive models. The XGBoost model had a better performance in both training and validation sets as compared with other models in terms of accuracy. Our data suggested that the XGBoost model is the most precise and personalized tool for predicting the 1-year survival rate for NSCLC patients with BM. This model can help the clinicians to design more rational and effective therapeutic strategies.

## 1. Introduction

Early-stage lung cancer is usually asymptomatic. Hence, lung cancer is frequently diagnosed at a late stage [1, 2]. Non-small-cell lung cancer (NSCLC) is the most common histological subtype of lung cancer, with about 40% of cases harboring distant metastases at the first diagnosis [3]. Bone metastases (BM) occurs in 30-40% of NSCLC patients, which is one of the most frequent distant metastasis events [4]. It is known that distant metastases are the leading cause of cancer-related death [5, 6]. For NSCLC patients with BM, the reported median survival is less than 1 year in different populations [7]. Such poor prognosis highlights the significant demand for accurate tools for predicting the prognosis of NSCLC with BM.

A TNM staging system is a tool based on pathological anatomy, which can assist clinicians to develop effective treatment strategies and improve the patients' prognosis [8]. However, the prognosis of patients with the same stage is notably different, indicating significant limitations of using the TNM staging system as the prognosis predicting model. More importantly, many other factors should be considered and involved in predicting the prognosis of patients [9, 10]. Survival prediction models designed for lung cancer with BM specifically have been reported previously [11–13]. However, the performance of these models is barely satisfactory as these models are based on the simple Cox regression model but not established as a survival prediction model for NSCLC with BM particularly. Given the impact of histological changes in prognostic determination, we propose narrowing down the scope of the study objects. For example, for patients with a certain histological type of NSCLC, it is necessary to improve the accuracy of the predictive model. Currently, artificial intelligence (AI) models based on machine learning (ML) algorithms are increasingly applied for clinical practice. Most models including random forest (RF), support vector machine (SVM), Bayesian network, and decision tree are created based on traditional ML algorithms [14]. Extreme gradient boosting (XGBoost) is a typical boosting algorithm designed to be highly efficient, flexible, and portable. Boosting is an ensemble technique with which new models can adjust the errors produced by existing models [15]. These advantages guarantee the high performance of XGBoost which provides satisfactory results in machine learning competitions and has been successfully used in other studies and domains [16].

Therefore, in the current study, we extracted the NSCLC patients with BM from the Surveillance, Epidemiology, and End Results (SEER) database and searched for an ideal AI model to predict the 1-year survival of NSCLC with BM by testing the XGBoost and other traditional algorithms.

## 2. Methods and Materials

### 2.1. Patients

All NSCLC patients with confirmed BM in the SEER database between 2010 and 2015 were selected for this study. The inclusion criteria were as follows: (a) patients diagnosed with lung cancer on histology, (b) the histologic type of NSCLC, and (c) patients with BM. The exclusion criteria were as follows: (a) lung cancer not the primary cancer, (b) patients without complete clinicopathological characteristics, demographic information, or follow-up information, and (c) follow-up time < 1 month at the follow-up deadline. Finally, we extracted 5973 NSCLC patients with BM from 309,056 lung cancer patients. The study population was distributed to the training and validation sets with a ratio of 7 : 3. The classification process was completely randomized, and it was performed in R software. In addition, we retrospectively collected data for NSCLC patients with BM from the Affiliated Hospital of Chengde Medical University (AHOCMU) between 2015 and 2019 as an external validation set for our research.

### 2.2. Data Collection

Based on the specific patient information available in the SEER database, we selected 19 variables that may affect the prognosis of NSCLC with BM, including age, sex, race, tumor size, tumor site, histological type, grade, laterality, surgery, chemotherapy, radiotherapy, TNM staging, distant metastasis sites (lung, brain, and liver), insurance status, and marital status.

The primary site is defined according to the International Classification of Diseases for Oncology (ICD-O) codes: main bronchus (C34.0), lobe (C34.1-C34.3), overlapping lesion of the lung (C34.8), and lung, if not otherwise specified (C34.9). The histological type is defined in accordance with the following ICD-O-3 codes: adenocarcinoma (8140, 8141, 8144, 8244, 8250–8255, 8260, 8290, 8310, 8323, 8333, 8470, 8480, 8481, 8490, 8507, 8550, 8551, 8570, 8571, 8574, and 8576), squamous cell carcinoma (8052, 8070-8076, 8078, 8083, 8084, and 8123), and other NSCLC (8004, 8012-8014, 8022, 8030, 8035, 8046, 8082, 8200, 8240, 8249, 8430, 8560, and 8562). Regarding marital status, we excluded the misleading data of unmarried or domestic partner, and then, “unmarried,” “separated,” “single,” and “widow” were all included in the unmarried group. The insurance status was divided into insured and uninsured; “any Medicaid,” “insured,” and “insured/no specific” were included in the insured group. All cases in the present study were staged using the 7th edition of the AJCC TNM staging system.

### 2.3. Prognostic Nomogram

The variables that might be related to prognosis were analyzed by the univariate analysis. Then, variables with *p* < 0.05 revealed by the univariate analysis were further included in the multivariate logistic analysis to determine independent prognostic factors of NSCLC patients with BM. Next, these independent prognostic factors identified by the multivariate logistic analysis were used to construct a nomogram for predicting the 1-year survival of NSCLC with BM.

### 2.4. Construction of the XGBoost Model

Before the sample feature data is put into the model for classification, the data was preprocessed first. In the dataset used in the study, age and size are continuous variables, and the rest is classified. For continuous, we adopted standardization for age and size to speed up the training. The formula is as follows:

(1) $$\begin{array}{c} x^{*}=\frac{x-\mu }{\sigma }. \end{array}$$

To calculate the distance accurately in some machine learning models, we used one-hot encoding for multiclassification variables. The tree-based model has an excellent performance to calculate the importance of features. XGBoost was used to rank feature importance, and eventually, significant variables were included in our model building. After variable selection, there were 17 feature variables left. We also used XGBoost, an ensemble machine learning method predicting the residuals of prior models, and then combined together to make the final prediction. XGBoost uses second-order Taylor series to estimate the value of the loss function and further reduces the likelihood of overfitting by application of regularization. The objective function is as follows:

(2) $$\begin{array}{c} L^{t}=\overset{n}{\underset{i=1}{\sum }}l\left(y_{i},{{\hat{y}}_{i}}^{t-1}+f_{t}\left(x_{i}\right)\right)+\Omega \left(f_{t}\right),\\ L^{t}≃\overset{n}{\underset{i=1}{\sum }}\left[l\left(y_{i},{\hat{y}}^{t-1}\right)+g_{i}f_{t}\left(x_{i}\right)+\frac{1}{2}h_{i}f_{1}^{2}\left(x_{i}\right)\right]+\Omega \left(f_{t}\right), \end{array}$$

where $g_{i}=\partial_{{\hat{y}}^{t-1}}l\left(y_{i},{{\hat{y}}_{i}}^{t-1}\right)$ and $h_{i}=\partial_{{\hat{y}}^{t-1}}^{2}l\left(y_{i},{{\hat{y}}_{i}}^{t-1}\right)$,

(3) $$\begin{array}{c} {\tilde{L}}^{t}=\overset{n}{\underset{i=1}{\sum }}\left[g_{i}f_{t}\left(x_{i}\right)+\frac{1}{2}h_{i}f_{1}^{2}\left(x_{i}\right)\right]+\Omega \left(f_{t}\right),\\ {\tilde{L}}^{t}=\overset{n}{\underset{i=1}{\sum }}\left[g_{i}f_{t}\left(x_{i}\right)+\frac{1}{2}h_{i}f_{t}^{2}\left(x_{i}\right)\right]+\gamma T+\frac{1}{2}\lambda \overset{T}{\underset{j=1}{\sum }}w_{j}^{2}=\overset{T}{\underset{j=1}{\sum }}\left[\left(\underset{i\in Ij}{\sum }g_{i}\right)w_{j}+\frac{1}{2}\left(\underset{i\in Ij}{\sum }h_{i}+\lambda \right)w_{j}^{2}\right]+\gamma T=-\frac{1}{2}\overset{T}{\underset{j=1}{\sum }}\frac{{\left(\sum_{i\in Ij}g_{i}\right)}^{2}}{\sum_{i\in Ij}h_{i}+\lambda }+\gamma T, \end{array}$$

where $\left[l\right.\left(y_{i},{{\hat{y}}_{i}}^{t-1}\right)$ is the loss function of time (*t* − 1), $\partial_{{\hat{y}}^{t-1}}l\left(y_{i},{\hat{y}}^{t-1}\right)$ is the partial derivative of the loss function time (*t* − 1), $\partial_{{\hat{y}}^{t-1}}^{2}l\left(y_{i},{\hat{y}}^{t-1}\right)$ is the second derivative of (*t* − 1) degree of loss function, and *Ω*(*f*_*t*_) is the complexity of model *f*(*t*). In the setup of the hyperparameters, the best values were determined by performing a grid search.

### 2.5. Model Evaluation

We have also established three other prediction models based on RF, SVN, and logistic algorithms, respectively. To evaluate the performance of each prediction model, receiver operating characteristic (ROC) curves were used to quantify and compare the predictive performance of the XGBoost model and other prediction models.

## 3. Results

### 3.1. Features of Patients

According to the inclusion and exclusion criteria, 5973 NSCLC patients with BM were selected from the SEER database, and an additional 114 NSCLC patients with BM were identified from the AHOCMU for this study. In addition, 4183 patients were enrolled in the training set; the rest 1790 patients were included in the validation set. Patient demographic and clinicopathologic features are presented in Table 1. Briefly, 4657 patients (78.0%) were white and 740 (12.4%) were black. Male (57.4%) had a slight predominance over female (42.6%). Regarding the tumor characteristics, 90.7% were located in the lung lobe; adenocarcinoma (64.5%) accounted for the majority, most of which were moderately or poorly differentiated. For therapy, 231 (3.9%) of the patients received surgery, 3853 (64.5%) received chemotherapy, and 3525 (59.0%) underwent radiotherapy. Lung metastases (28.6%) were more common than liver metastases (20.4%) and brain metastases (23.1%).

### 3.2. Prognostic Nomogram for 1-Year Survival

**Figure 1:**
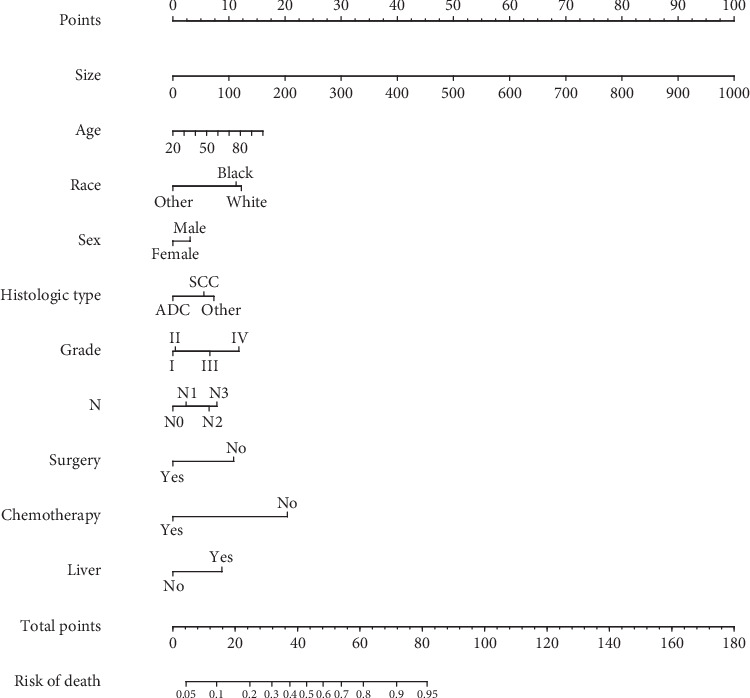
Nomogram to predict the 1-year survival of NSCLC with BM.

The univariate analysis is presented in Table 2. The results of the multivariate logistic analysis indicated that tumor size, age, race, sex, histological type, grade, N stage, surgery, chemotherapy, and liver metastases were OS-related prognostic factors (Table 2). Next, these prognostic factors were integrated to build a nomogram for predicting the prognosis of NSCLC with BM (Figure 1). As shown in Figure 1, tumor size is the most important prognostic factor followed by chemotherapy, age, race, grade, surgery, and liver metastases, which affected the prognosis moderately, while N stage, histologic type, and sex had little effect on prognosis. Furthermore, each prognostic factor was given a corresponding score for the nomogram. The total score was obtained by summing the scores of each relevant factor, and we used the total score to draw a vertical line to obtain the individual probability of NSCLC with BM survival.

### 3.3. Establishment of the XGBoost Model

**Figure 2:**
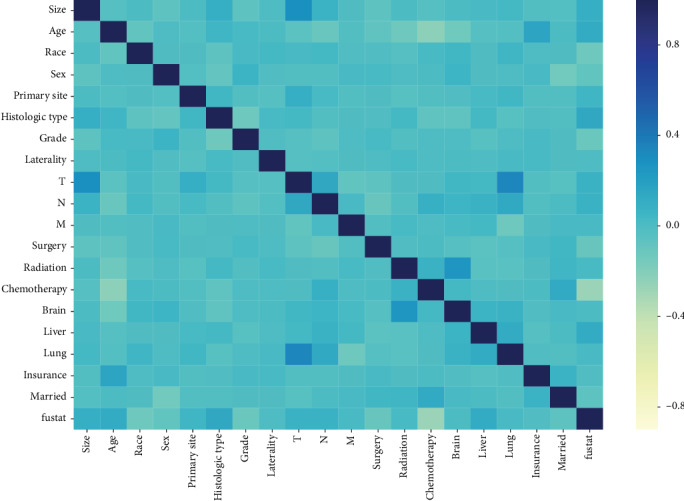
Heatmap of pair correlations. In dark blue are statistically positive significant correlations; in light yellow statistically significant inverse correlations, and in light blue not statistically significant correlations.

**Figure 3:**
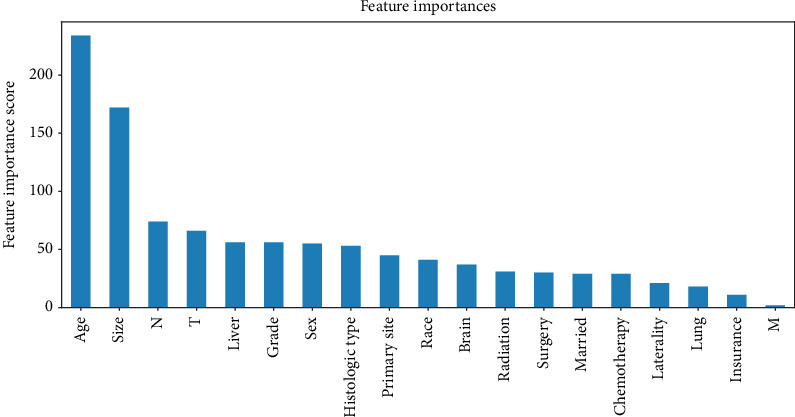
Results of feature importance for using XGBoost. The bar plots present the exact number of times the top features are selected.

Correlated features are redundant and may decrease the performance of ML algorithms. The correlations between the features are depicted in Figure 2. Thus, it was necessary to perform the feature reduction. The nineteen features were ranked using the XGBoost Classifier based on feature importance. The ranking is shown in Figure 3. A cut-off point was determined to select the top-ranked features for the best trade-off between model performance and simplicity, according to the accuracy of the model when using different thresholds. After this selection, the M stage and insurance status were removed, and 17 features were fitted into our model.

**Figure 4:**
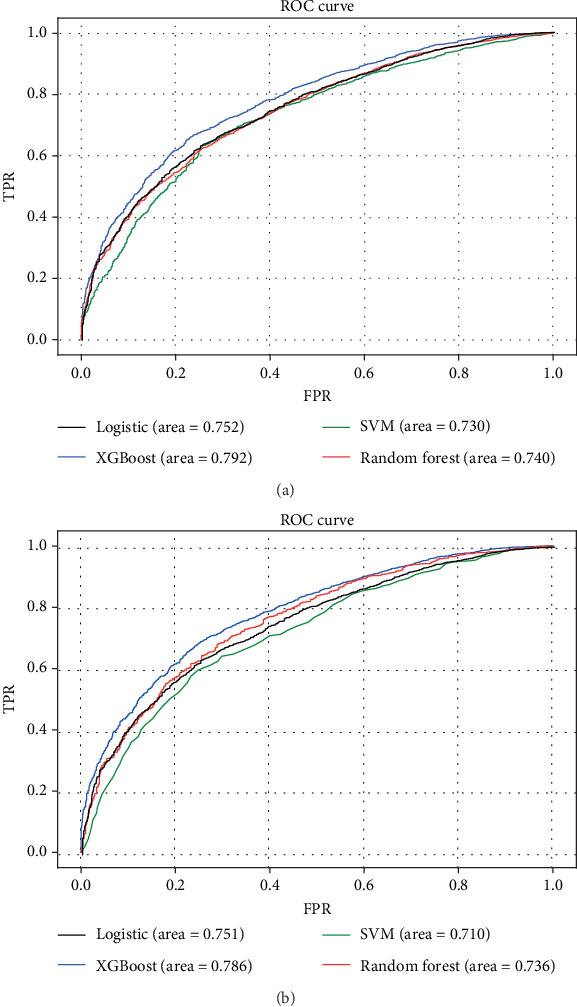
ROC curves showing the predictions of the four models: XGBoost, SVM, RF, and logistic. (a) The training set; (b) the internal validation set.

After grid search, the parameters of the best model were determined (learning_rate = 0.15, max_depth = 3, min_child_weight = 3, subsample = 0.9, colsample_bytree = 0.7, n_estimators = 40, scale_pos_weight = 1, and nthread = −1). Using ROC analysis, the prediction model using XGBoost achieved a fitted AUC of 0.792 in the training set (Figure 4).

### 3.4. Validation of Predictive Accuracy of the XGBoost Model

**Figure 5:**
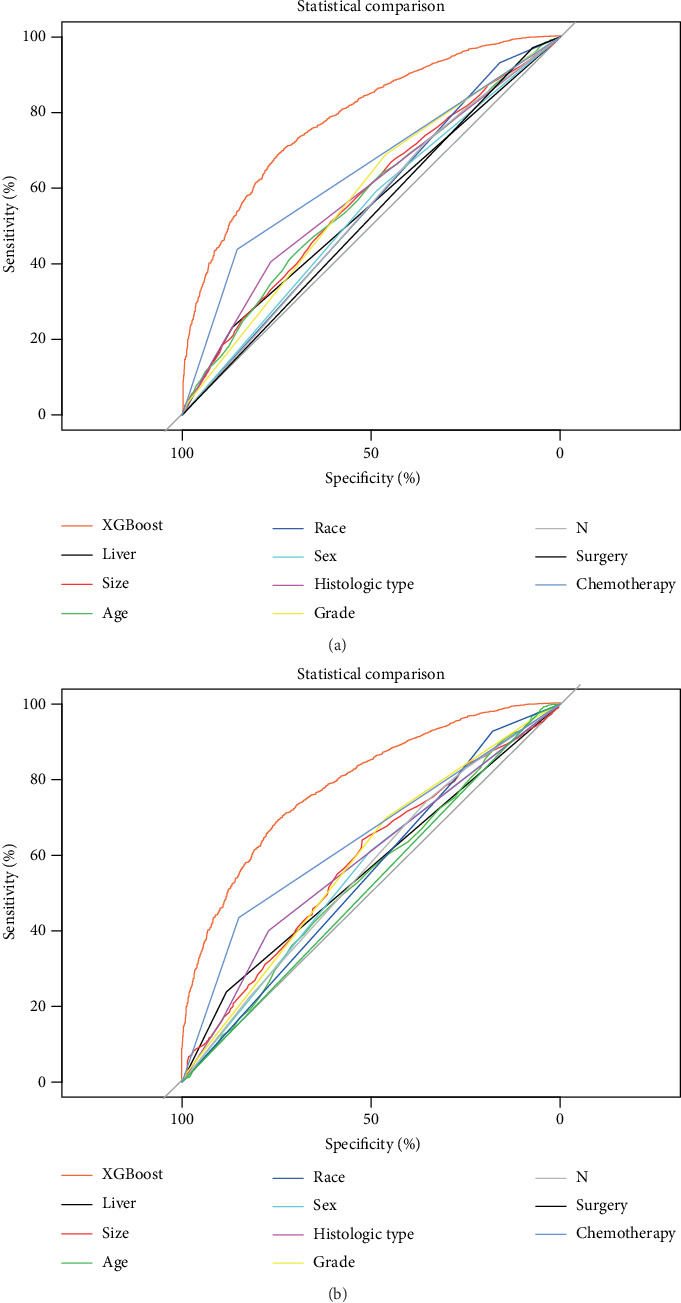
Comparison of prediction accuracy between XGBoost model and independent prognostic factor. (a) The training set; (b) the validation set.

**Figure 6:**
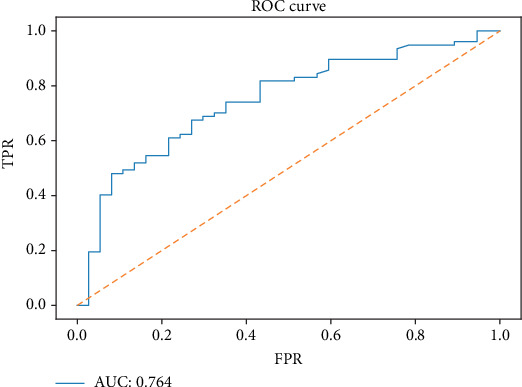
ROC curve of the XGBoost model in predicting the 1-year survival of NSCLC with BM in the external validation set.

We depicted the ROC curves for the XGBoost model and the single prognostic factor in training and validation sets, respectively. As shown in Figure 5, the AUC of the XGBoost model was significantly bigger than the single prognostic factor, indicating a much higher prediction accuracy of the XGBoost model. At the same time, the XGBoost model had an AUC of 0.764 in the external validation set, demonstrating a better discriminative ability (Figure 6).

### 3.5. Comparison of Predictive Accuracy between Various Prediction Models

In order to assess the advantage of the prediction model generated by the XGBoost algorithm, we also compared it with other models. The training and validation sets of each model were depicted with ROC curves, and the corresponding AUCs were calculated. In the training set, the accuracy of the XGBoost model for predicting survival (AUC = 0.792) was higher than that of RF (AUC = 0.740), SVM (AUC = 0.730), and logistic (AUC = 0.752) (Figure 4(a)). The XGBoost model also had a better performance in the validation set (AUC = 0.786), compared with RF (AUC = 0.736), SVM (AUC = 0.710), and logistic (AUC = 0.751) (Figure 4(b)).

## 4. Discussion

Although NSCLC with BM may obtain longer survival than before with advancement of various treatment methods and drugs, accurate prediction of survival for NSCLC with BM remains to be necessary and a challenge for clinicians. This study established and validated the XGBoost model as the most appropriate model for predicting 1-year survival of NSCLC with BM. In essence, the XGBoost model achieved an AUC of 0.792, 0.786, and 0.764 in the training, internal validation, and external validation sets, respectively. Compared with other models, it showed better reliability and accuracy (Figure 4), which could be utilized to predict the 1-year mortality of NSCLC with BM, thus facilitating a reasonable individualized drug treatment program determination.

To our knowledge, this study is the first research to establish a prognostic model for NSCLC with BM by using AI-based models on large-scale populations. The major differences between our study and the others could be summarized as follows. First, we scaled down the research objects to NSCLC instead of the entire lung cancer patient population. It was important as the histological subtype affected the prognosis dramatically, which was in line as previously reported [17–19]. Second, our study only included patients with BM, but not with other metastases, as the prognosis of patients with different metastatic sites was quite different [20, 21]. Therefore, the accuracy of using a prognostic model based on patients with any metastatic NSCLC to predict the prognosis of NSCLC with BM was questionable. More importantly, most of those previous predictive models were based on the Cox regression model or logistic regression. Logistic regression and Cox regression are regular algorithms that can be replaced by more sophisticated algorithms. For instance, XGBoost has excellent performance for processing large-scale and high-dimensional data [22]. Taken together, after defining NSCLC patients with BM, we then constructed and validated a prediction model based on the XGBoost algorithm, avoiding the shortcomings of the other models, and achieved the best performance in prediction among all models (Figure 2).

According to our study, tumor size is a significant factor affecting the patient prognosis, which has not been recognized previously [11–13]. T stage roughly classifies tumor size or depth of invasion, but it cannot reflect the specific character of NSCLC patients with BM and accurately predict the prognosis, because the tumor size of the same period varies greatly [23–25]. Age, tumor size, race, sex, histological types, grade, T stage, N stage, surgery, chemotherapy, liver metastases, and radiotherapy were related to prognosis, which was similar to the previous research [26–30]. The presence of liver metastases significantly decreased survival in lung cancer patients with BM [17]. Firstly, it may be related to the liver being an immunosuppressive organ, thus hindering the immune surveillance of the growing metastases of the liver; secondly, the worse response to chemotherapy caused by metastatic liver cancer leads to a worse prognosis [31]. Undifferentiated, late-stage patients did show worse prognosis as expected, which is consistent with general cognition. We also find that surgery and chemotherapy are generally beneficial for patients. However, due to the lack of specific surgical procedures, chemotherapy-specific drugs, and specific programs, we were unable to further explore the relationship between treatment methods and prognosis in more depth. Of note, molecular targeted therapy and immunotherapy may provide new options in addition to traditional surgery, radiotherapy, and chemotherapy; whether these new treatments affect NSCLC with BM requires further investigation although some recent studies show that epidermal growth factor receptor-targeted drugs can improve prognosis in lung cancer [31, 32].

Although deep learning is accessible in the academics and industry, the boosting algorithm based on a tree model still plays a significant role in some subjects, showing dominance in structured information. The boosting algorithm has been proved to be effective in the predictive model of classification and regression tasks in practice. Traditional regression model and machine learning methods, such as Cox regression and SVM, are limited in terms of learning capacities, which need many artificial feature engineering. The tree-based model is a nonlinear model, which has the advantages of a natural feature combination and strong feature expressive capacity [33]. Tree-based classifiers, including RF and XGBoost, based on homogeneity, fit the characteristics of the data set for the present study. We speculated that the application of regularization, using Taylor expansion to estimate the loss function, and high flexibility to allow for fine-tuning might enable XGBoost to perform better than RF [34]. Taken together, our findings suggested that the XGBoost approach can reflect the feature importance and set up a mortality prediction model with extreme accuracy. Furthermore, this approach has extreme potential for practical implementation because it can be incorporated into existing healthcare information systems.

Our research had some advantages. First, the SEER database provided complete follow-up information of patients covering a large scope. Second, our AI model could provide personalized survival prediction for patients, thereby improving personalized treatment. Finally, our AI model can be used to predict the survival of other NSCLC patients with BM, as all the information used to predict survival is easily accessible, and our model can be performed as software-based or web-based tool optimization. However, this study has certain limitations. First, this study was a retrospective study while prospective randomized clinical trials are needed to provide high-level evidence for clinical application. Second, we could not obtain specific information about the treatment, such as chemotherapy drugs and protocols, radiation doses, and specific surgical procedures.

## 5. Conclusion

We used the XGBoost algorithm to build an AI model that predicts the 1-year survival of NSCLC with BM. The XGBoost model has higher accuracy and better performance than models generated from other algorithms. Furthermore, the XGBoost model can be integrated into existing healthcare information systems, so we propose that the XGBoost model could be used as a practical clinical prediction model to help clinicians develop better and more reasonable treatment programs.

## Figures and Tables

**Table 1:** Demographic and clinicopathologic features of 5973 NSCLC patients with BM in the SEER database.

Variables	Training set	Validation set	*t*/*χ*^2^	*p*
Age (mean ± SD)	66.41 ± 11.01	66.51 ± 10.92	0.336	0.737
Size (mean ± SD)	51.79 ± 34.44	52.20 ± 33.81	0.423	0.673
Race			1.191	0.551
Black	519 (12.4%)	221 (12.3%)		
Other	392 (9.4%)	184 (10.3%)		
White	3272 (78.2%)	1385 (77.4%)		
Sex			0.146	0.702
Female	1789 (42.8%)	756 (42.2%)		
Male	2394 (57.2%)	1034 (57.8%)		
Primary site			0.350	0.950
Main bronchus	173 (4.1%)	79 (4.4%)		
Overlapping lesion of lung	31 (0.7%)	12 (0.7%)		
Lung, NOS	182 (4.4%)	76 (4.2%)		
Lobe	3797 (90.8%)	1623 (90.7%)		
Histologic type			6.010	0.050
ADC	2688 (64.3%)	1163 (65.0%)		
Others	558 (13.3%)	269 (15.0%)		
SCC	937 (22.4%)	358 (20.0%)		
Grade			2.367	0.500
I	217 (5.2%)	86 (4.8%)		
II	1259 (30.1%)	536 (29.9%)		
III	2596 (62.1%)	1131 (63.2%)		
IV	111 (2.7%)	37 (2.1%)		
Laterality			0.277	0.599
Left—origin of primary	1750 (41.8%)	762 (42.6%)		
Right—origin of primary	2433 (58.2%)	1028 (57.4%)		
T stage			5.837	0.120
T1	446 (10.7%)	184 (10.3%)		
T2	1168 (27.9%)	555 (31.0%)		
T3	1139 (27.2%)	469 (26.2%)		
T4	1430 (34.2%)	582 (32.5%)		
N stage			3.715	0.294
N0	887 (21.2%)	343 (19.2%)		
N1	373 (8.9%)	170 (9.5%)		
N2	2039 (48.7%)	902 (50.4%)		
N3	884 (21.1%)	375 (20.9%)		
M stage			0.916	0.339
M1a	110 (2.6%)	55 (3.1%)		
M1b	4073 (97.4%)	1735 (96.9%)		
Radiotherapy			1.270	0.260
No	1734 (41.5%)	714 (39.9%)		
Yes	2449 (58.5%)	1076 (60.1%)		
Chemotherapy			0.099	0.753
No	1490 (35.6%)	630 (35.2%)		
Yes	2693 (64.4%)	1160 (64.8%)		
Surgery			1.452	0.228
No	4013 (95.9%)	1729 (96.6%)		
Yes	170 (4.1%)	61 (3.4%)		
Brain metastasis			2.261	0.133
No	3239 (77.4%)	1354 (75.6%)		
Yes	944 (22.6%)	436 (24.4%)		
Liver metastasis			0.002	0.960
No	3330 (79.6%)	1426 (79.7%)		
Yes	853 (20.4%)	364 (20.3%)		
Lung metastasis			0.576	0.448
No	2999 (71.7%)	1266 (70.7%)		
Yes	1184 (28.3%)	524 (29.3%)		
Insurance status			1.036	0.309
Insured	4059 (97.0%)	1728 (96.5%)		
Uninsured	124 (3.0%)	62 (3.5%)		
Marital status			0.099	0.753
Married	2412 (57.7%)	1040 (58.1%)		
Unmarried	1771 (42.3%)	750 (41.9%)		

NSCLC: non-small-cell lung cancer; BM: bone metastasis; ADC: adenocarcinoma; SCC: squamous cell carcinoma.

**Table 2:** Univariate analysis and multivariate logistic analysis based on all variables for 1-year survival (training cohort).

Characteristics	Univariate analysis	Multivariate logistic analysis	
	*p* value	HR (95% CI)	*p* value
Age	<0.001	1.015 (1.008–1.022)	<0.001
Size	<0.001	1.008 (1.004–1.011)	<0.001
Race			
Black	<0.001	Reference	
Other		0.423 (0.311–0.575)	<0.001
White		1.073 (0.851–1.352)	0.552
Sex			
Female	<0.001	Reference	
Male		1.265 (1.088–1.470)	<0.05
Primary site			
Main bronchus	<0.05		
Overlapping lesion of lung			
Lung, NOS			
Lobe			
Histologic type			
ADC	<0.001	Reference	
Others		1.746 (1.351–2.255)	<0.001
SCC		1.524 (1.246–1.865)	<0.001
Grade			
I	<0.001	Reference	
II		1.037 (0.751–1.433)	0.824
III		1.653 (1.206-2.266)	<0.05
IV		2.454 (1.292-4.659)	<0.05
Laterality			
Left—origin of primary	0.752		
Right—origin of primary			
T stage			
T1	<0.001		
T2			
T3			
T4			
N stage			
N0	<0.001	Reference	
N1		1.198 (0.901-1.592)	0.215
N2		1.636 (1.351-1.982)	<0.001
N3		1.816 (1.443-2.284)	<0.001
M stage			
M1a	0.791		
M1b			
Radiotherapy			
No	0.162		
Yes			
Surgery			
No	<0.001	Reference	
Yes		0.438 (0.311-0.617)	<0.001
Chemotherapy			
No	<0.001	Reference	
Yes		0.211 (0.174 -0.256)	<0.001
Brain metastasis			
No	0.866		
Yes			
Liver metastasis			
No	<0.001	Reference	
Yes		1.948 (1.589-2.388)	<0.001
Lung metastasis			
No	0.949		
Yes			
Insurance status			
Insured	0.732		
Uninsured			
Marital status			
Married	<0.001		
Unmarried			

ADC: adenocarcinoma; SCC: squamous cell carcinoma.

## Data Availability

The datasets generated and/or analyzed during the current study are available in the SEER database (https://seer.cancer.gov/).

## Abbreviations

NSCLC: Non-small-cell lung cancer
BM: Bone metastases
AI: Artificial intelligence
ML: Machine learning
RF: Random forest
SVM: Support vector machine
XGBoost: Extreme gradient boosting
SEER: Surveillance, Epidemiology, and End Results
ROC: Receiver operating characteristic
AUC: Area under the curve.
